# Narrative strategies and interaction performance in Olympic short videos on Chinese mainstream media: evidence from the 2024 Paris Summer Olympics

**DOI:** 10.3389/fpsyg.2026.1825366

**Published:** 2026-04-28

**Authors:** Gengxin Yi, Haojie Wang

**Affiliations:** Guangdong Medical University, Dongguan, China

**Keywords:** audience engagement, emotional narratives, Olympic games, short-video platforms, sports communication

## Abstract

Short-video platforms have become an important arena for the dissemination of mega sporting events and have significantly reshaped the organization and circulation of Olympic-related content. In this communication context, the content structure and narrative strategies of mainstream media’s Olympic short videos provide an important entry point for understanding audience interaction. This study examines 219 Olympic-related short videos published by the CCTV News Douyin account during the 2024 Paris Summer Olympic Games. Using quantitative content analysis, it analyzes the content structure of these videos in terms of domestic relevance, narrative focus, highlight moments, and emotional narratives, and further investigates the relationship between different narrative strategies and interaction visibility, measured by likes, comments, and shares. The results show that Olympic short videos exhibit a strong domestic relevance orientation and a clear athlete-centered narrative structure, while highlight moments and emotional narratives are widely adopted in narrative expression. However, highlight moments do not show significant differences across interaction indicators, and the relationships between emotional narratives and interaction performance vary across likes, comments, and shares. By comparison, narrative focus shows a more stable association with audience interaction. Further analysis shows that different combinations of narrative strategies were associated with significant differences in likes, comments, and shares. These findings provide new empirical evidence for understanding the content production logic and audience interaction mechanisms of mainstream media’s Olympic short videos in the short-video era.

## Introduction

For a long time, the Olympic Games mainly relied on TV broadcasting to reach the global audience (Billings, 2008), but limited by the length of broadcasting and channel resources, the audience has limited access to the event content (Scott et al., 2024; Cummins and Hahn, 2022). In the era of mobile Internet, the form of Olympic communication has undergone a fundamental change: social media and short video platforms make Olympic content more dynamic and multi-dimensional, and audience participation and consumption patterns also change (Triantafyllou et al., 2025). In this sense, short video is not only the migration of broadcasting channels, but the reorganization of the Olympic narrative. The narrative unit has changed from “the whole event/the whole report” to “the narrative segments that can be consumed and re-disseminated quickly”, and the narrative focus is more likely to focus on the characters’ moments, key plots, and emotional expression (Mou et al., 2025).

During the 2024 Paris Summer Olympic Games, the short video communication mode was embedded in the core link of Olympic Games communication with unprecedented depth. In China, the daily activity of users of the short video platform is as high as 800 million, and the video broadcasting volume related to the 2024 Paris Summer Olympic Games is as high as 100 billion times, which makes the Olympic Games communication of mainstream media from the “program layout logic” to the “platform narrative logic” competition field (National Radio and Television Administration Development Research Center et al., 2023, p. 18). Unlike TV reports, which emphasize time allocation, event broadcasting and highlights editing, short videos emphasize “instant presentation of visual situations” and “rapid release of emotional intensity.” Under the guidance of the interactive mechanism, the meaning production of reports is put in front of the selection of content structure and narrative strategies (Cummins and Hahn, 2022). Therefore, the key to understanding the short video reports of the 2024 Paris Summer Olympic Games is not whether they are reported, but how the mainstream media organize the Olympic significance through the content structure and narrative strategies in the platform context, and what kind of interaction visibility differences it presents. Emotional storytelling is widely considered an effective strategy for attracting audience attention and stimulating engagement on digital platforms. However, whether emotional narratives actually enhance interaction in short-video sports communication remains an open empirical question.

Around the narrative characteristics of Olympic reports, the academic community has accumulated rich research results. Most of the existing studies are from the perspective of social identity, framework theory and nationalism to explore the narrative distribution and presentation form in the report, as well as the differences in the nationality, gender and race of athletes (Capranica et al., 2005; Yoon, 2013; Delorme and Testard, 2015; Sakaki et al., 2022; MacArthur and Smith, 2021; Yang et al., 2023; Scott et al., 2024). A large number of studies have shown that the media often focus on the narrative weight of “relevant objects in the country”, and complete the meaning framing in the narrative boundary of “we/them” (Xu et al., 2017; Scott et al., 2019; MacArthur and Smith, 2021; Scott et al., 2024). At the same time, individualized narration, highlighting plots and emotional expression are regarded as one of the most efficient ways of organizing Olympic narratives. They can strengthen the character and emotional input in a limited information window, and then shape the audience’s understanding of the event (Cummins and Hahn, 2022; Scott et al., 2024). In recent years, research on Chinese media sports narrative has gradually increased, which provides an important reference for explaining the content selection and significance construction of mainstream media in major events (Li et al., 2022; Zeng and Yang, 2024; Li et al., 2025; Mou et al., 2025).

However, compared with the research on the macro communication effect of TV broadcast or social media, there is still a lack of systematic testing on the relationship between the narrative structure and the interaction performance of the mainstream media on the short video platform. During the 2024 Paris Summer Olympic Games, CCTV’s new media matrix released 2,105 Olympic-related products, with a total broadcast of more than 13.19 billion times (China Media Group General Manager’s Office, 2024). This shows that short video has become an important position for the mainstream media to spread the Olympic Games. But how does the mainstream media construct the Olympic narrative in the context of short videos? Does its content structure continue the characteristics of the TV era? What is the relationship between narrative strategies and communication effect? These questions have not been answered systematically. Although the existing research has provided a foundation for cross-media and transnational comparison, there is still a lack of special investigation into short video platforms (Xu et al., 2018; Scott et al., 2024; Zhang and Liao, 2025). This study extends previous Olympic reporting research beyond television-centered analysis by focusing on the narrative logic and combination effects of Olympic short videos produced by a major Chinese mainstream media account on Douyin. In doing so, it offers an empirical perspective on how narrative strategies operate in this specific platform-based communication context.

## Literature review

### Theoretical foundations

When analyzing the content structure and narrative strategies of Olympic short videos produced by mainstream media, agenda-setting theory and framing theory provide two closely related analytical perspectives. Agenda-setting theory suggests that the media influence public perceptions of “what is important” by highlighting specific issues and objects (McCombs and Shaw, 1972). In the networked communication environment, this perspective has been further extended to the construction of associations among issues, objects, and attributes, as emphasized in network agenda-setting theory (Guo and McCombs, 2016).

Framing theory, by contrast, focuses on how media select, emphasize, and organize information to construct meaning, thereby shaping how audiences interpret and evaluate events (Goffman, 1974; Tudor, 1992; Entman, 1993; Entman, 2007). In journalism studies, frames are often manifested through the arrangement of specific plot elements, modes of expression, and interpretive pathways, all of which influence how audiences understand social events (Nelson et al., 1997). In the context of sports communication, prior research has also shown that event coverage is not merely informational but often relies on narrative strategies to construct meaning and emotional orientation (Cummins and Hahn, 2022; Mou et al., 2025).

Existing studies on Olympic communication indicate that media coverage tends to highlight particular subjects—such as domestic athletes, national delegations, or key competitive contexts—while organizing content and meaning through character-centered narratives, key moments, and emotional expression (MacArthur and Smith, 2021; Scott et al., 2024; Cummins and Hahn, 2022). This suggests that both content structure and narrative expression serve as important entry points for understanding media representation in Olympic short videos.

Based on this, agenda-setting theory helps explain which objects and themes are made salient, while framing theory helps explain how these elements are organized and expressed. In this study, the former mainly corresponds to salience distribution in content structure, while the latter corresponds to meaning construction in narrative expression.

### Distributional characteristics of Olympic short video content

In major sports event coverage, media content is typically not evenly distributed. A substantial body of research shows that media across countries tend to emphasize domestic athletes and national delegations in Olympic reporting, reflecting a common pattern of national relevance in sports media narratives (Billings et al., 2013; MacArthur and Smith, 2021; Scott et al., 2024). For example, Delorme and Testard (2015) found that domestic athletes were more prominent both in frequency and importance in French Olympic coverage.

In the Chinese context, previous studies have also shown that major sports coverage often strengthens the symbolic meaning of domestic athletes through character-centered and honor-based narratives, thereby contributing to the construction of collective identity and national image (Li et al., 2022; Zeng and Yang, 2024). However, these studies have primarily focused on television broadcasts, traditional news texts, or general social media content. Systematic empirical analysis of Olympic short videos produced by mainstream media on short-video platforms remains limited.

In addition to reporting objects, sports coverage also exhibits relatively stable patterns in narrative organization. Prior research suggests that Olympic coverage tends to focus more on individual athletes rather than evenly distributing attention across delegations, event processes, or peripheral information (Capranica et al., 2005; Delorme and Testard, 2015). Such individualized narratives provide clearer character-driven storylines and recognizable narrative entry points (Scott et al., 2024). In the short-video environment, this tendency may be further reinforced due to time constraints and accelerated pacing (Mou et al., 2025).

In terms of narrative expression, highlight moments and emotional expression are also commonly used resources in Olympic coverage. Previous studies indicate that sports communication often enhances narrative tension by presenting key competitive moments such as victories, comebacks, and record-breaking performances, while emotional cues help strengthen audience immersion and affective engagement (Cummins and Hahn, 2022; Sakaki et al., 2022; Yang et al., 2023; Mou et al., 2025). Moreover, the short-video platform emphasizes immediacy, visual impact, and emotional appeal, making highlight moments and emotional narratives particularly salient and observable features (Triantafyllou et al., 2025).

Overall, existing studies suggest that Olympic coverage may exhibit distinct distributional patterns in terms of national relevance, narrative focus, and expressive strategies. However, these findings are largely based on television or general social media contexts. Whether such patterns are reproduced or intensified in mainstream media Olympic short videos on short-video platforms remains to be examined. Therefore, this study first proposes:

*RQ1*: What distributional characteristics are evident in the content structure and narrative strategies of Olympic short videos produced by Chinese mainstream media during the 2024 Paris Summer Olympics?

### Narrative focus and interaction visibility

In sports communication research, narrative focus is considered an important factor shaping how content is understood. Prior studies indicate that athlete-centered narratives are more likely to provide clear character-driven storylines compared to narratives centered on event processes or general information (Capranica et al., 2005; Delorme and Testard, 2015; Scott et al., 2024). Such narratives are also more capable of incorporating elements such as growth, competition, and emotion (Cummins and Hahn, 2022; Mou et al., 2025).

In the short-video context, character-centered narratives offer an additional advantage: they can quickly establish a narrative focal point within a limited time frame. Audiences do not need to process extensive background information but can directly engage with clearly defined characters and storylines. Based on this, it is reasonable to expect that athlete-centered narratives may be more likely to elicit visible audience interaction compared to event-centered or peripheral content. In this study, interaction visibility refers to observable engagement behaviors such as likes, comments, and shares.

It should be noted that existing studies mainly suggest that character-centered narratives enhance storytelling, immersion, and recognizability, but have not directly confirmed that they necessarily improve interaction performance in the specific context of Olympic short videos on Douyin. Therefore, the following hypothesis is proposed as a theoretically and empirically informed expectation:

*H1*: Athlete-centered narratives are associated with higher interaction visibility compared to event-centered or peripheral narratives.

### Highlight moments and interaction visibility

Highlight moments typically refer to key points in a competition, such as victories, comebacks, or record-breaking performances. Prior research suggests that such content possesses strong visual appeal and narrative intensity, making it a central component of sports storytelling (Cummins and Hahn, 2022; Sakaki et al., 2022). In Olympic coverage, highlight moments often serve not only to convey information but also to structure narrative climax and emotional rhythm.

From a communication perspective, highlight moments are highly salient and can quickly capture audience attention, which aligns with the short-video platform’s emphasis on rapid engagement (Triantafyllou et al., 2025). Therefore, it is reasonable to expect that videos containing highlight moments may be more likely to generate immediate audience responses.

However, existing studies mainly document the prevalence and appeal of highlight moments rather than providing consistent evidence that they increase interaction across different indicators in this specific platform context. Accordingly, the following hypothesis is proposed:

*H2*: Videos containing highlight moments are associated with higher interaction visibility than those without highlight moments.

### Emotional narratives and interaction visibility

Emotional expression has long been recognized as an important narrative resource in sports communication. Prior research suggests that emotions such as joy, disappointment, excitement, and frustration can enhance the affective appeal of content and may increase audience engagement (Yang et al., 2023; Mou et al., 2025). In social media and short-video environments, emotional cues often become highly salient features of content (Triantafyllou et al., 2025).

From this perspective, emotional narratives may increase the likelihood of visible audience interaction by enhancing emotional involvement. In other words, there is a theoretical basis for expecting a positive association between emotional expression and interaction performance.

However, this relationship should not be assumed to be overly strong. In the short-video context, emotional expression may already be widely used; when emotional elements are highly prevalent, their ability to differentiate interaction performance may be reduced. In addition, if emotional narratives are operationalized in a simplified or binary manner, their explanatory power may be constrained. Therefore, while a positive expectation is proposed, the strength of this relationship remains an empirical question.

*H3*: Videos containing emotional narratives are associated with higher interaction visibility than those without emotional narratives.

### Combinations of narrative strategies and interaction visibility

It should be further noted that narrative elements in short-video content rarely appear in isolation. A single video may simultaneously include multiple elements, such as athlete-centered narratives, highlight moments, and emotional expression. While prior studies have examined sports communication from perspectives such as content features, emotional expression, or audience engagement, relatively little attention has been paid to how these elements jointly shape interaction outcomes (Triantafyllou et al., 2025; Mou et al., 2025).

Analyzing individual variables helps determine whether a specific narrative strategy is associated with interaction visibility. However, it does not fully explain whether different combinations of narrative elements lead to differentiated interaction patterns. Therefore, in addition to examining individual effects, this study further explores the combined effects of narrative strategies.

*RQ2*: Do different combinations of narrative strategies lead to differentiated patterns of interaction visibility?

## Methods

### Research design and samples

Using quantitative content analysis, this study examines the reporting structure and narrative strategies of Chinese mainstream media on the 2024 Paris Summer Olympic Games in the context of short videos. The sample selected the Olympic-related short videos released by the CCTV News Douyin account during the Games (26 July–11 August 2024). After platform retrieval and manual review, duplicate, irrelevant, and incomplete videos were excluded, resulting in 219 valid samples with a total length of 164 min and 39 s.

The account was selected as the research object because of its national mainstream media positioning, agenda-setting capacity, and strong communication influence. It provides a relevant case for examining how Olympic short videos are organized and narrated by a major Chinese mainstream media actor on Douyin. The research takes a single short video as the analysis unit, and integrates video screen, subtitles, dubbing and interview content for overall analysis.

### Coding framework and variable operation

In line with the theoretical framework of this study, domestic relevance orientation and narrative focus were treated as variables reflecting the salience structure of Olympic short-video reporting, whereas highlight moments and emotional narrative were treated as variables reflecting narrative framing strategies.

The construction of the coding framework is based on the core logic of “research problem adaptability + existing literature support”, combined with the short video “emotional, individualized and scene-based” communication characteristics, referring to the content analysis paradigm of the media Olympic report in the field of sports communication (Billings and Angelini, 2007; Scott et al., 2024). At the same time, it focuses on the core relationship between content characteristics, narrative strategies and communication effects, and finally determines the three-dimensional coding framework to ensure that variables can accurately respond to research issues.

(1) Core narrative dimensions (four items): (a) domestic relevance orientation (0 = non-China, 1 = Chinese foreign mix, 2 = China-led); (b) narrative focus (1 = individual athlete, 2 = national team/delegation, 3 = event/off-field content); (c) highlight moments (0 = absent, 1 = present); and (d) emotional narrative (0 = absent, 1 = present). For emotional narrative, coders assessed three subdimensions: emotional language, emotional audiovisual treatment, and explicit evaluative judgment. A video was coded as containing emotional narrative when at least two of these three indicators were present; otherwise, it was coded as 0.

(2) Narrative situation structure (one auxiliary variable): key plot type, including six typical plot categories such as finals/medal-winning moments and record-breaking scenes; this variable was used only for descriptive scenario analysis.

(3) Interaction visibility indicators (three items): number of likes, number of comments, and number of shares, based on the platform-displayed counts of each video. These indicators were used to capture visible forms of audience interaction in the short-video environment.

### Coding process and reliability test

In this study, three coders completed the coding work together. The coders have certain communication research experience, and received a unified research program and coding rules training before the official coding (Scott et al., 2024). Each coder first encoded the initial samples, accounting for about 15% of the total data set. After encoding, the chief researcher checked the coding results of the three coders, and calculated the coder to coder reliability using the formula of Cohen (1960), and determined the reliability of the following variables: (a) domestic relevance orientation [K = 0.892]; (b) the narrative focus [K = 0.876]; (c) highlight moments [K = 0.810]; (d) emotional narrative [K = 0.898]; (e) interaction visibility index [K = 1.00]. The coder-to-coder reliability calculated by Cohen’s Kappa coefficient exceeded 0.80 in all categories, reaching an acceptable coder-to-coder reliability level (Riffe et al., 2023). These scores show that researchers have good consistency and reliability in encoding short video content to corresponding categories. After the reliability test and unified correction of coding differences, each coder independently codes the remaining samples.

### Data processing and analysis strategy

To address RQ1, descriptive statistics were conducted for domestic relevance orientation, narrative focus, highlight moments, and emotional narrative. Chi-square goodness-of-fit tests were then used to examine whether the distributions of the main variables deviated significantly from a uniform structure. To test H1–H3, nonparametric tests were employed because the interaction indicators (likes, comments, and shares) showed significantly skewed distributions. Specifically, the Mann–Whitney U test was used for two-category variables, and the Kruskal–Wallis H test was used for three-category variables. Accordingly, the analysis concerns differences in interaction visibility across videos. To address RQ2, the study further examined combinations of narrative strategies in order to identify possible patterns of interaction visibility associated with different narrative configurations.

## Results

### The content structure characteristics of Olympic-related short videos

To address RQ1, this section examines the content structure of Olympic-related short videos from four aspects: reporting subject, narrative organization, narrative expression, and narrative situation. Specifically, through the five variables of domestic relevance orientation, narrative focus, highlight moments, emotional narrative and key plot types, descriptive statistics were conducted on 219 Olympic Games short video samples, and the chi square goodness of fit test was used to investigate whether the category distribution of the main variables was significantly deviated from the uniform structure.

At the main level of the report, this study conducts statistical analysis on the “domestic relevance orientation” variables. As shown in Table 1, of the 219 Olympic Games short video samples, there were 201 Chinese leading content, accounting for 91.78%. There were 8 mixed contents, accounting for 3.65%. There were 10 non-Chinese leading contents, accounting for 4.57%. The results of the chi square goodness of fit test show that the category distribution of domestic relevance orientation significantly deviates from the theoretical uniform distribution of three categories accounting for the same proportion, *χ*^2^(2, N = 219) = 336.69, *p* < 0.001. According to Cohen (1988) effect quantity calculation method, Cohen’s w = 1.24 is obtained, and the effect quantity reaches a maximum level, indicating that the Olympic short video shows a highly concentrated domestic related structure in the reporting subject.

**Table 1:** Distribution of domestic relevance in Olympic-related short videos.

Category	Frequency (*n*)	Percentage (%)
China-led content	201	91.78
Mixed China–foreign content	8	3.65
Non-China-led content	10	4.57
Total	219	100.00

At the narrative organization level, this study further analyzes the distribution of “narrative focus.” As shown in Table 2, there are 159 videos focusing on athletes, accounting for 72.60%; 15 videos focused on the national team or delegation, accounting for 6.85%; 45 videos focused on event information or off-site content, accounting for 20.55%. Chi square goodness of fit test showed that the category distribution of narrative focus also significantly deviated from the uniform structure, *χ*^2^ (2, N = 219) = 158.14, *p* < 0.001, Cohen’s *w* = 0.85, The effect amount reached a maximum level. The results show that the narrative organization of Olympic-related short videos shows a clear “individualized” orientation, that is, the narrative is centered on the athletes themselves.

**Table 2:** Distribution of narrative focus in Olympic-related short videos.

Category	Frequency (*n*)	Percentage (%)
Individual athlete	159	72.60
National team/delegation	15	6.85
Event information or off-field content	45	20.55
Total	219	100.00

At the level of narrative expression, this study makes a statistical analysis of the two variables of “highlight moments” and “emotional narrative.” As shown in Table 3, of the 219 samples, 158 videos contained highlights, accounting for 72.15%; There were 61 videos without highlights, accounting for 27.85% Chi square test results showed that the distribution was significantly deviated from the uniform structure, *χ*^2^(1, *N* = 219) = 42.96, *p* < 0.001, Cohen’s *w* = 0.44, The effect amount reached a large level. In terms of emotional narration, 211 videos including emotional expression accounted for 96.35%, whereas 8 videos did not, accounting for 3.65%. Chi square test results show that the distribution is significantly deviated from the uniform structure, χ^2^ (1, *N* = 219) = 188.17, *p* < 0.001, Cohen’s *w* = 0.93. The effect amount reached a maximum level.

**Table 3:** Distribution of highlight moments and emotional narrative.

Variable	Category	Frequency (*n*)	Percentage (%)
Highlight moments	Present	158	72.15
	Absent	61	27.85
Emotional narrative	Present	211	96.35
	Absent	8	3.65

At the narrative situation level, this study makes descriptive statistics on the key plot types. As shown in Table 4, the most common plot type is the final or medal-winning plot (*n* = 145,66.21%), followed by record-breaking or historical first-time plot (*n* = 47,21.46%). The occurrence frequency of the plot against a strong enemy (*n* = 26,11.87%) and the key turning plot (*n* = 15,6.85%) is relatively low, while injury or withdrawal (*n* = 7,3.20%) and controversial penalty or conflict (*n* = 1,0.46%) are less common in the sample.

**Table 4:** Types of highlight moments in Olympic-related short videos.

Type of highlight moment	Frequency (*n*)	Percentage (%)
Final/medal-winning moment	145	66.21
Record-breaking/historic first	47	21.46
Key turning point/comeback	15	6.85
Facing strong opponents	26	11.87
Injury/withdrawal	7	3.20
Controversial judgment/conflict	1	0.46

In general, in the context of short video communication, the mainstream media Olympic short video shows three significant characteristics in content structure. The main body of the report is highly concentrated on the relevant objects in the country. The narrative organization is centered on the individual athletes. The narrative expression generally adopts highlight presentation and emotional expression, and the plot selection mainly focuses on the key event nodes.

### Narrative strategies and interaction visibility

To test H1–H3, this study further examined the relationship between different narrative strategies and interaction visibility. Interaction visibility was measured by the number of likes, comments and shares. Since all three indicators showed clearly skewed distributions, nonparametric tests were used for the analysis.

First, the mean ranks of different narrative categories across the interaction indicators were calculated, as shown in Table 5. From the descriptive results, different narrative strategies have some differences in the performance of interaction. For example, in terms of narrative focus, the average rank of the athlete-centered narrative in the likes and comments index is relatively high, while the average rank of the event information or off-site content centered narrative in the three interaction indicators is relatively low.

**Table 5:** Interaction visibility across narrative strategies (mean ranks).

Narrative strategy	Category	*N*	Mean rank (likes)	Mean rank (comments)	Mean rank (shares)
Domestic relevance	China-led	201	110.75	110.14	108.20
	Mixed China–foreign	8	88.00	100.81	116.63
	Non-China-led	10	112.50	114.60	140.95
Narrative focus	Individual athlete	159	115.60	118.12	113.54
	National team/delegation	15	118.53	110.67	133.70
	Event/off-field content	45	87.38	81.08	89.59
Highlight moments	Absent	122	108.43	102.48	106.70
	Present	97	110.61	112.90	111.28
Emotional narrative	Absent	16	126.94	147.50	155.25
	Present	203	109.36	108.58	108.28

Then, nonparametric tests were performed on all variables, and the results are summarized in Table 6. Domestic relevance orientation did not reach a statistically significant level in likes, comments, or shares. Narrative focus showed significant differences across all three interaction indicators, indicating that videos with different narrative centers varied significantly in interaction visibility. This result provides support for H1. By contrast, highlight moments showed no significant differences in likes, comments, or shares, and therefore H2 was not supported. Emotional narrative did not reach a significant level in likes or comments, but showed a significant difference in shares. Accordingly, H3 was only partially supported. In general, among the single narrative dimensions examined in this study, narrative focus showed the most consistent association with interaction visibility, whereas highlight moments and emotional narrative showed less consistent patterns.

**Table 6:** Nonparametric tests of interaction visibility across narrative strategies.

Independent variable	Dependent variable	Test method	Statistic	*p*-value	*P*
Domestic relevance (three groups)	Likes	Kruskal–Wallis *H*	*H* = 1.01	0.604	—
Domestic relevance (three groups)	Comments	Kruskal–Wallis *H*	*H* = 0.22	0.895	—
Domestic relevance (three groups)	Shares	Kruskal–Wallis *H*	*H* = 2.64	0.268	—
Narrative focus (three groups)	Likes	Kruskal–Wallis *H*	*H* = 7.25	0.027	*
Narrative focus (three groups)	Comments	Kruskal–Wallis *H*	*H* = 11.99	0.002	**
Narrative focus (three groups)	Shares	Kruskal–Wallis *H*	*H* = 7.27	0.026	*
Highlight moments (two groups)	Likes	Mann–Whitney *U*	*U* = 4723.00	0.819	—
Highlight moments (two groups)	Comments	Mann–Whitney *U*	*U* = 4360.50	0.275	—
Highlight moments (two groups)	Shares	Mann–Whitney *U*	*U* = 4617.50	0.632	—
Emotional narrative (two groups)	Likes	Mann–Whitney *U*	*U* = 979.50	0.441	—
Emotional narrative (two groups)	Comments	Mann–Whitney *U*	*U* = 1144.00	0.088	—
Emotional narrative (two groups)	Shares	Mann–Whitney *U*	*U* = 1206.00	0.040	*

*p < 0.05; ** *p* < 0.01; *** *p* < 0.001.

### Effect analysis of narrative strategies combination

Building on the tests of H1–H3, this study further addresses RQ2 by examining how combinations of multiple narrative elements are associated with interaction visibility. Because short-video narratives usually contain more than one narrative element, a single variable alone cannot fully capture the relationship between content structure and communication performance.

In this study, narrative focus, highlight moments and emotional narrative are used to construct narrative strategies combination variables, forming nine types of narrative strategies combination. Among them, “athlete + highlight moments + emotional narrative” is the most common narrative structure in the sample (*n* = 132), followed by “event/off-site content + no highlight + emotional narrative” (*n* = 29) and “athlete + no highlight + emotional narrative” (*n* = 27). The frequency of other narrative combinations is relatively low.

See Table 7 for the average rank distribution of interaction indicators of different narrative strategies combinations. From the descriptive results, there are some differences in the average rank of each narrative structure in the likes, comments and forwarding indicators. However, due to the small sample size of some combinations, the average rank results are mainly used for descriptive reference.

**Table 7:** Interaction visibility across narrative strategy combinations (mean ranks).

Narrative focus	Highlight moments	Emotional narrative	*N*	Mean rank (likes)	Mean rank (comments)	Mean rank (shares)
1	0	1	27	138.63	137.19	139.52
1	1	1	132	110.89	114.22	108.23
2	0	1	3	95.83	80.33	85.17
2	1	0	1	111.00	119.00	140.00
2	1	1	11	125.41	118.18	146.36
3	0	0	2	157.50	162.00	179.00
3	0	1	29	78.22	68.36	73.38
3	1	0	5	117.90	147.40	148.80
3	1	1	9	84.33	67.22	89.06

In order to test whether these differences are statistically significant, this study uses Kruskal Wallis *H* test for comparison. The test results show that different narrative strategies combinations have statistically significant differences in the number of likes (*H* = 16.31, *p* = 0.038), comments (*H* = 26.13, *p* = 0.001) and forwards (*H* = 25.19, *p* = 0.001) (see Table 8).

**Table 8:** Kruskal–Wallis tests for narrative strategy combinations.

Interaction indicator	Test method	Number of groups (k)	df	*H*	*p*-value
Likes	Kruskal–Wallis *H*	9	8	16.31	0.038
Comments	Kruskal–Wallis *H*	9	8	26.13	0.001
Shares	Kruskal–Wallis *H*	9	8	25.19	0.001

These results indicate that interaction visibility varied across different combinations of narrative strategies in the sampled Olympic short videos.

## Discussion

### Chinese mainstream media still focus on highlighting domestic content in Olympic-related short videos

This study found that the content structure of short Olympic videos released by Chinese mainstream media during the 2024 Paris Summer Olympic Games showed a very obvious domestic relevance orientation. China’s dominant content accounted for 91.78%, much higher than non-Chinese content. This shows that even if the communication platform is transferred from TV to short video, the Olympic Games coverage is still focused on highlighting domestic content. This finding is basically consistent with the conclusions of existing studies. The media of various countries tend to focus on their athletes and delegations (Billings and Eastman, 2002; Delorme and Testard, 2015; Scott et al., 2024). This reporting structure is considered to be closely related to the construction of national identity in sports communication research (MacArthur and Smith, 2021). The results of this study show that this structure is not weakened by the change of media form, but continues to maintain stability and further strengthen on the short video platform.

However, compared with the television era, the Olympic Games short video shows some new characteristics. In traditional TV reports, the advantages of China are usually reflected through the broadcast time, lens allocation or comment context. However, on the short video platform, this tendency is more reflected in the content selection, such as narrating around the key moments, character stories or post-game emotional expression of domestic athletes. In other words, domestic relevance orientation is not only a distribution structure of reported objects, but also an important situational entry for short video narration.

### Characterization narrative becomes the core organization of short video report

At the narrative organization level, the Olympic short video of China’s mainstream media obviously presents an athlete-centered narrative focus. In all the samples, 72.60% of the videos focus on the individual athletes, while the content centered on the national team or the whole event is significantly less. This result is consistent with the classic findings in sports media research. Many studies have pointed out that sports reports tend to organize the content through character stories, because character narration is easier to form a plot structure and emotional connection (Cummins and Hahn, 2022; Scott et al., 2024). Athletes’ growth experiences, competition moments or post-competition reactions can provide clear narrative lines for events. However, on the short video platform, this personalised narrative seems to be further strengthened. On the one hand, the length of short videos is limited, so it is necessary to establish situations and emotions in a very short time, and character narration can quickly form the story framework. On the other hand, platform algorithms are often easier to recommend content with emotional tension or personal stories. Therefore, in the context of short video, narrating around athletes is not only a way of reporting, but also an important strategy to improve communication efficiency.

This finding provides support for H1 and suggests that athlete-centered storytelling is more likely to generate visible audience response than event-centered or off-field narratives in the short-video context.

### Highlight moments and emotional narratives are common, but their interaction advantages are not consistent

At the level of narrative expression, this study finds that both highlight moments and emotional narratives are widely used in Olympic short videos. In particular, emotional narratives account for a substantial proportion of the sample, suggesting that emotional expression has become a central narrative resource in short-video sports communication. This finding is consistent with prior research emphasizing the importance of emotional expression in sports media (Mou et al., 2025).

However, the hypothesis testing results indicate that highlight moments and emotional narratives do not function in the same way in relation to interaction performance. First, H2 is not supported. The results show that highlight moments do not produce significant differences across any of the three interaction indicators (likes, comments, and shares). This suggests that although highlight moments are commonly used in Olympic short videos, they do not translate into a stable interaction advantage in the current sample.

Second, H3 is only partially supported. Emotional narratives are significantly associated with shares, but not with likes or comments. This indicates that the relationship between emotional narratives and interaction performance is not uniform across different types of user engagement. More cautiously, it can be argued that, under the current sample and measurement conditions, emotional narratives do not demonstrate a consistent advantage across interaction indicators, but instead show a heterogeneous pattern of association.

Importantly, this finding does not imply that emotional narratives are ineffective in short-video communication. On the contrary, their high prevalence in the sample suggests that they have become a routine narrative resource in Olympic short video production. Precisely because emotional cues are so widely present, emotional narratives may function more as a “baseline configuration” than as a distinguishing feature that differentiates levels of interaction performance. In such a context, the mere presence of emotional expression may have limited explanatory power for variation in interaction outcomes.

In addition, emotional narratives are operationalized as a binary variable in this study, which facilitates coding reliability but inevitably compresses variations in emotional type, intensity, and narrative function. Different emotions (e.g., excitement, inspiration, disappointment, or pride) may elicit different forms of audience response, yet such distinctions are not captured in the current measurement. Therefore, the role of emotional narratives in Olympic short videos is better understood as conditional and context-dependent, rather than as a uniformly effective strategy.

By contrast, narrative focus shows a more stable association with interaction visibility. This suggests that, in short-video environments, how content is organized—particularly whether it centers on individual athletes—may provide a more consistent basis for differentiating interaction performance than the mere presence of highlight moments or emotional expression.

### The influence of narrative strategies combination on interaction visibility

Different combinations of narrative strategies showed significant differences across likes, comments, and shares in the sampled videos. This pattern indicates that the interaction performance of Olympic short videos was associated not only with single narrative features, but also with the way multiple narrative elements were combined within specific video texts. In the context of short-video communication, narrative focus, highlight moments, and emotional narrative do not appear in isolation; rather, they are organized together within particular narrative structures. From this perspective, the observed differences in interaction visibility point to the analytical value of examining Olympic short-video narratives in configurational terms.

### Theoretical contributions and research limitations

Based on Olympic-related short videos published by Chinese mainstream media, this study empirically examined the content characteristics and narrative strategies of Olympic reporting in the short-video context, thereby providing an empirical perspective on the communication mechanisms of mainstream media’s Olympic short videos. At the same time, several limitations should be acknowledged. First, the sample was drawn from a single mainstream media account on a single platform, and the findings should therefore be understood within the specific institutional and platform context of CCTV News on Douyin. In particular, CCTV News operates within a Chinese mainstream media environment that differs from many commercial or more pluralized media systems in terms of institutional role, agenda-setting logic, and platform use, while Douyin’s platform ecology, recommendation mechanisms, and user culture may shape short-video circulation in ways that are not fully comparable to other platform settings. Second, audience response was measured through platform-visible interaction indicators—likes, comments, and shares—which reflect interaction visibility but do not fully account for potential differences in exposure across videos. Third, although emotional narrative was coded through a composite rule, its final measurement remained relatively general, as the variable was reduced to a binary form and did not distinguish emotional valence, intensity, or narrative function. In addition, the high prevalence of emotional narrative in the sample further limited variation in the data. Future research could employ more differentiated indicators, broader samples, and comparative designs to further examine interaction patterns in Olympic short-video communication across different media environments.

## Conclusion

This study finds that Olympic short videos produced by Chinese mainstream media during the 2024 Paris Summer Olympics exhibit clear distributional patterns: China-related content dominates, athlete-centered narratives are most prevalent, and both highlight moments and emotional narratives are widely used. The results further show that narrative focus demonstrates the most stable association with interaction performance across likes, comments, and shares. By contrast, highlight moments do not show significant differences, and emotional narratives do not exhibit consistent patterns across interaction indicators. In addition, different combinations of narrative strategies are associated with differentiated interaction performance, indicating that audience engagement is shaped not only by individual elements but also by their configurations within short-video content.

## Data Availability

The raw data supporting the conclusions of this article will be made available by the authors, without undue reservation.
